# Differential binding of chemokines CXCL1, CXCL2 and CCL2 to mouse glomerular endothelial cells reveals specificity for distinct heparan sulfate domains

**DOI:** 10.1371/journal.pone.0201560

**Published:** 2018-09-24

**Authors:** J. J. van Gemst, M. Kouwenberg, A. L. W. M. M. Rops, T. H. van Kuppevelt, J. H. Berden, T. J. Rabelink, M. A. Loeven, J. van der Vlag

**Affiliations:** 1 Department of Nephrology, Radboud Institute for Molecular Life Sciences, Radboud university medical center, Nijmegen, The Netherlands; 2 Department of Biochemistry, Radboud Institute for Molecular Life Sciences, Radboud university medical center, Nijmegen, The Netherlands; 3 Department of Nephrology and Einthoven Laboratory for Vascular Medicine, Leiden University Medical Center, Leiden, The Netherlands; University of South Alabama Mitchell Cancer Institute, UNITED STATES

## Abstract

**Introduction:**

Proliferative glomerulonephritis manifests in a range of renal diseases and is characterized by the influx of inflammatory cells into the glomerulus. Heparan sulfate (HS) is an important (co-)receptor for binding of chemokines, cytokines and leukocytes to the endothelial glycocalyx, a thick glycan layer that covers the inside of blood vessels. During glomerulonephritis, HS in the glomerular endothelial glycocalyx plays a central role in chemokine presentation and oligomerization, and in binding of selectins and integrins expressed by leukocytes. We hypothesize that distinct endothelial HS domains determine the binding of different chemokines. In this study we evaluated the interaction of three pro-inflammatory chemokines (CXCL1, CXCL2 and CCL2) with mouse glomerular endothelial cells (mGEnC-1) in ELISA in competition with different HS preparations and anti-HS single chain variable fragment (scFv) antibodies specific for distinct HS domains.

**Results:**

HS appeared to be the primary ligand mediating chemokine binding to the glomerular endothelial glycocalyx *in vitro*. We found differential affinities of CXCL1, CXCL2 and CCL2 for HS in isolated mGEnC-1 glycocalyx, heparan sulfate from bovine kidney or low molecular weight heparin in competition ELISAs using mGEnC-1 as a substrate, indicating that chemokine binding is affected by the domain structure of the different HS preparations. Blocking of specific HS domains with anti-HS scFv antibodies revealed a domain-specific interaction of the tested chemokines to HS on mGEnC-1. Furthermore, chemokines did not compete for the same binding sites on mGEnC-1.

**Conclusion:**

CXCL1, CXCL2 and CCL2 binding to the glomerular endothelial glycocalyx appears differentially mediated by specific HS domains. Our findings may therefore contribute to the development of HS-based treatments for renal and possibly other inflammatory diseases specifically targeting chemokine-endothelial cell interactions.

## Introduction

Glomerulonephritis is one of the main renal disease manifestations worldwide and can lead to end stage renal disease and kidney failure. The majority of proliferative forms of glomerulonephritis display a massive influx of leukocytes into the glomerulus, i.e. the filtration compartment of the kidney [1, 2]. Trafficking of leukocytes on activated endothelium involves the concerted action of cytokines, chemokines, adhesion molecules and glycosaminoglycans (GAGs) [3–8]. Chemokines play a central role in inflammation as intercellular signalling molecules that direct cell migration towards sites of chemokine production by interacting with chemokine receptors on target cells [9]. Inflammatory processes in glomerulonephritis strongly depend on the pro-inflammatory chemokines CXCL8 (interleukin-8 (IL-8)), CCL2 (monocyte chemotactic protein-1 (MCP-1)) and CXCL2 (macrophage inflammatory protein-2α (MIP-2α)) to direct neutrophils and macrophages towards the glomerulus [10–13]. GAGs, the main structural components of cell surface glycan layers, including the glomerular endothelial glycocalyx, are pivotal in glomerular inflammation [7, 14, 15] because they function as (co-) receptors for chemokines to establish a local chemokine concentration gradient. Binding of chemokines to GAGs: i) can enhance presentation of chemokines to their corresponding receptor, ii) facilitate chemokine oligomerization and activity and iii) protect chemokines against proteolysis, thereby conserving chemotactic stimuli for prolonged periods of time [16–19].

GAGs are long linear, negatively charged polysaccharides, and GAGs in the endothelial glycocalyx include heparan sulfate (HS), chondroitin sulfate (CS) and non-sulfated hyaluronan (HA). HS is the most structurally heterogeneous GAG in the endothelial glycocalyx. HS chains are covalently attached to the cell surface via a core protein, and are involved in various biological processes, including cell adhesion, signaling, migration and proliferation. A HS chain consists of up to 200 repeating disaccharide units of β(1–4)-N-acetyl glucosamine (GlcNAc)-α(1–4)-glucuronic acid (GlcA) and can be modified extensively. Modifications include N-sulfation, 6-O sulfation and 3-O sulfation of GlcNAc, 2-O sulfation of GlcA, as well as C5-epimerisation of GlcA to iduronic acid (IdoA). The resulting structural heterogeneity of HS forms the molecular basis for its broad specificity for various proteins and function in numerous pathways [14].

Using anti-HS single chain variable fragment (scFv) antibodies we have previously identified specific HS domains that are increasingly expressed on glomerular endothelial cells after activation by inflammatory stimuli *in vitro* and *in vivo* [6, 7, 20, 21]. Furthermore, we found that endothelial cell-specific knockout of N-deacetylase/N-sulfotransferase-1, an enzyme required for N-deacetylation and N-sulfation of HS, significantly reduced glomerular endothelial chemokine and leukocyte binding, thereby decreasing the inflammatory response in mice with experimentally induced anti-glomerular basement membrane nephritis [22]. While the majority of basic chemokines bind to the negatively charged GAGs, interactions appear to be at least partially specific [17]. Furthermore, certain acidic chemokines, such as MIP-1β, interact with HS/heparin despite their overall negative charge [19]. We therefore hypothesize that distinctly modified endothelial HS domains determine the binding of different chemokines during inflammation. We investigated the role of HS in binding of the chemokines CXCL1 (Groα/KC) and CXCL2 (Mip-2α), which are functional murine IL-8 homologues [23–26], and CCL2 (MCP-1) to mouse glomerular endothelial cells (mGEnC-1) *in vitro*. CXCL1, CXCL2 and CCL2 were found to exhibit specific and dose-dependent binding to mGEnC-1 that was mediated by distinct HS domains, suggesting that chemokine-specific HS analogs could be applied to interfere with inflammation in the kidney and possibly other organs.

## Materials and methods

### Mouse glomerular endothelial cell culture and treatment with HS-degrading enzymes

Conditionally immortalized mouse glomerular endothelial cells (mGEnC-1) with all features of primary mouse glomerular endothelial cells, including expression of glycocalyx components, have been developed in our lab in the past and were cultured as described [27]. Where indicated, cells were activated by incubation with 10 ng/ml tumor necrosis factor (TNF)-α (Life Technologies Europe, Bleiswijk, The Netherlands) for 18 hours. HS was digested using a combination of 0.25 U/ml heparinase I, II and III (Sigma-Aldrich Chemie, Zwijndrecht, The Netherlands) in 100 mM sodium acetate, 0.2 mM calcium acetate (pH 7.0) for 1 hour at 37°C. Removal of HS was confirmed with the scFv anti-HS antibody AO4B08 [28].

### Binding of CXCL1, CXCL2 and CCL2, and scFv anti-HS antibodies to mouse glomerular endothelial cells in ELISA

For evaluation of chemokine binding, confluent monolayers of TNF-α-activated mGEnC-1 in 96-well plates (Corning Life Sciences, Amsterdam, The Netherlands) were pre-treated for 10 min at room temperature with 100 μg/ml heparin (Sigma-Aldrich Chemie) in phosphate-buffered saline (PBS) to remove proteins bound to cell surface HS and washed extensively to remove residual heparin. Recombinant murine chemokines CXCL1, CXCL2 and CCL2 (Prospec-Tany, TechnoGene, Rehovot, Israel) were diluted serially (0–5 μg/ml) in 0.5% (w/v) polyvinyl alcohol (PVA) (Sigma-Aldrich Chemie) in PBS as blocking agent and incubated for 30 minutes at room temperature [29]. Bound chemokines were detected using the biotinylated polyclonal antibodies anti-mouse CXCL1 (BAF-453) (R&D systems, Minneapolis, USA), anti-mouse CXCL2 (AAM48B) (Bio-Rad, Veenendaal, The Netherlands) and anti-mouse CCL2 (505907) (Biolegend, San Diego, USA), followed by peroxidase-conjugated streptavidin (Thermo Scientific, Rockford, USA). Finally, the cells were washed with PBS and incubated with tetramethylbenzidine (TMB) solution (ITK Diagnostics, Uithoorn, The Netherlands). After 15 minutes, the reaction was stopped with 2M H_2_SO_4_ and absorbance at 450 nm was measured using a Bio-Rad Multiplate Reader (Bio-Rad). VSV-G-tagged scFv anti-HS antibodies used to block chemokine binding to cell surface HS are listed in Table 1. Binding of scFv antibodies was detected using a peroxidase-conjugated anti-VSV-G antibody (Sigma-Aldrich Chemie).

**Table 1:** Characteristics of HS domains recognized by anti-heparan sulfate (HS) scFv antibodies.

Antibody	V_H_—CDR3*	HS modifications required for antibody binding:	References
**AO4B08**	SLRMNGWRAHQ	N-sulfation, 6-O sulfation, 2-O sulfation, C5-epimerization	[28]
**EW3D10**	GRTVGRN	N-sulfation, 6-O sulfation	[30]
**EW4G2**	GKVKLPN	N-sulfation, 6-O sulfation, Glucuronic acid	[30]
**HS4C3**	GRRLKD	N-sulfation, 6-O sulfation, 3-O sulfation, 2-O sulfation	[31]
**HS3A8**	GMRPRL	N-sulfation, 6-O sulfation, 2-O sulfation, C5-epimerization	[28]
**HS4E4**	HAPLRNTRTNT	N-sulfation, N-acetylation, C5-epimerization	[28]
**LKIV69**	GSRSSR	N-sulfation, 2-O sulfation, C5-epimerization	[32]
**RB4Ea12**	RRYALDY	N-sulfation, N-acetylation, 6-O sulfation	[28]
**EV3C3**	GYRPRF	N-sulfation, 2-O-sulfation, C5-epimerization	[28]

*Given are the amino acid sequences of the heavy chain variable region complementarity-determining region 3 (VH—CDR3).

### Flow cytometry

mGEnC-1 were detached with 10 mM ethylenediaminetetraacetic acid (EDTA) in PBS and washed. The cells were incubated with 5 μg/ml recombinant mouse CXCL1, CXCL2 or CCL2 (Prospec-Tany) in 0.5% PVA for 1 hour at room temperature. Subsequently, cells were incubated with the biotinylated anti-chemokine antibodies described above for 30 minutes at 4°C, followed by Alexa 488-labeled streptavidin (Invitrogen Life Technologies, Breda, The Netherlands). VSV-tagged scFv antibodies were detected using a monoclonal mouse anti-VSV IgG_1_ antibody (clone P5D4; Sigma-Aldrich Chemie) followed by an Alexa 488-labeled goat anti-mouse IgG (H+L) antibody (Invitrogen, Life Technologies). Fluorescence was measured using a cytomics FC 500 flow cytometer and analyzed using CXP software (Beckman Coulter).

### Isolation of mGEnC-1-derived glycocalyx and agarose gel electrophoresis

mGEnC-1 glycocalyx was isolated and analyzed using barium acetate agarose gel electrophoresis as described [33]. For analysis, 0.5–1 μg of isolated GAGs were diluted 6x in 50 mM barium acetate, pH 5.0 electrophoresis buffer containing 20% glycerol, 0.01% bromophenol blue and separated on 1% multipurpose agarose gels in electrophoresis buffer. Where indicated, mGEnC-1 glycocalyx was treated with 0.25 U/ml each of heparinase I, II and III or chondroitinase ABC (Sigma-Aldrich Chemie).

### Anti-HS antibody/GAG/chemokine competition for CXCL1, CXCL2 and CCL2 binding to mouse glomerular endothelial cells

TNF-α-activated mGEnC-1 cells were grown in 96-well plates and washed with PBS. The effect of blocking specific HS domains on chemokine binding was determined by pre-incubation of mGEnC-1 with anti-HS antibodies (25 μg/ml) in 0.5% PVA for 30 minutes at room temperature. For competition experiments between two different chemokines, cells were pre-incubated with the competing chemokine for 30 minutes at room temperature. The effect of GAG competition on chemokine binding to mGEnC-1 was determined by pre-incubation of 750 ng/ml recombinant mouse CXCL1, CXCL2 or CCL2 with 0 to 100 μg/mL of isolated mGEnC-1 glycocalyx, HS from bovine kidney (HSBK) or enoxaparin before adding the chemokines to the cells. Cells were washed with PBS and bound chemokines were detected as described above.

### Statistical analysis

All values are expressed as means ± s.e.m. and significance between groups was determined by student t-test, or ANOVA, with Tukey's post-hoc test for multiple comparison using Graphpad Prism, version 5.0 software (Graphpad software, Inc., San Diego, USA). Experiments were performed at least in triplicate.

## Results

### HS mediates CXCL1, CXCL2 and CCL2 binding to mouse glomerular endothelial cells *in vitro*

**Fig 1:**
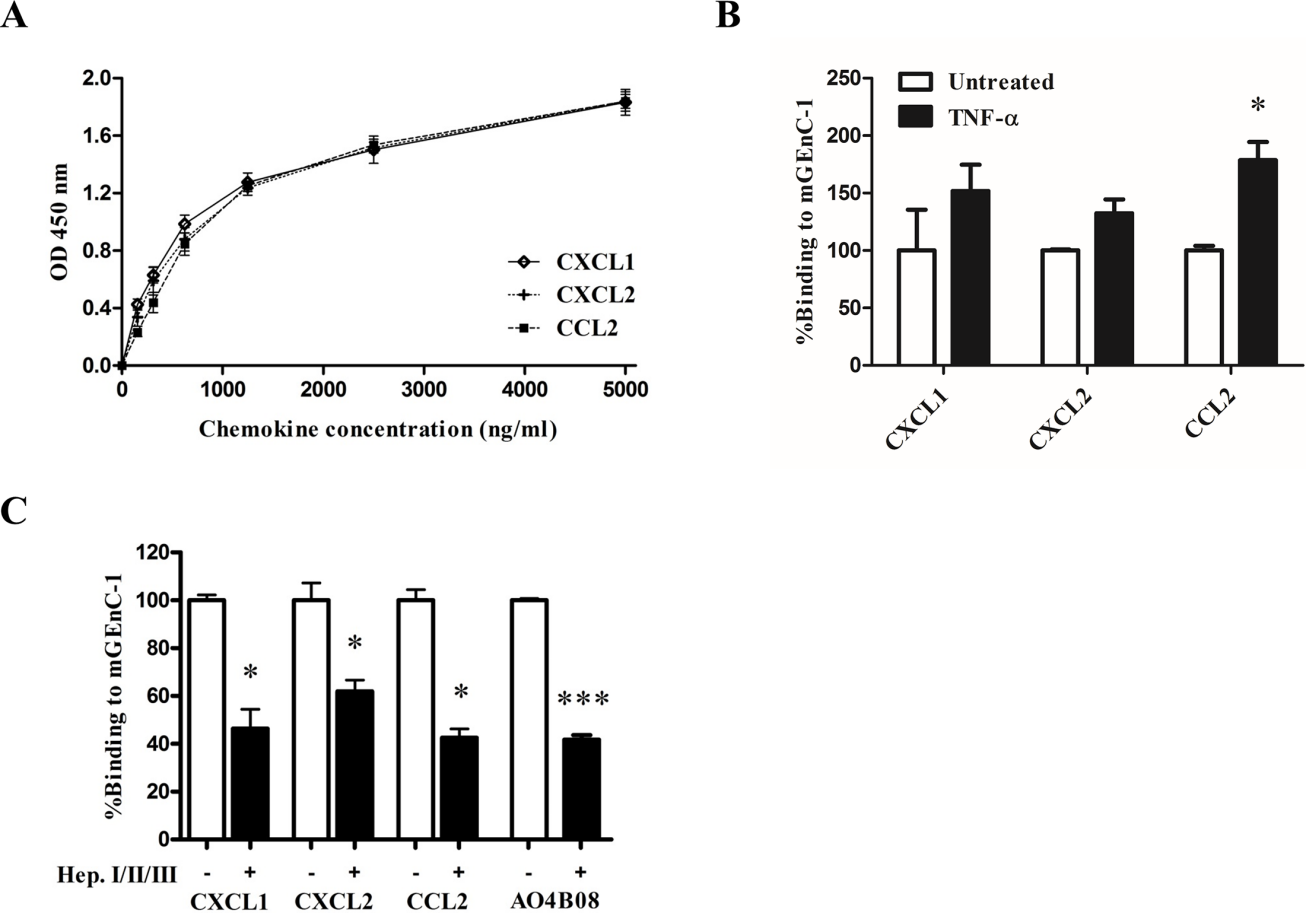
CXCL1, CXCL2 and CCL2 binding to mGEnC-1 is mediated by heparan sulfate (HS). Serial dilutions of mouse recombinant chemokines CXCL1, CXCL2 and CCL2 were added to TNF-α-activated mouse glomerular endothelial cells (mGEnC-1) and binding was detected using ELISA (A). CXCL1, CXCL2 and CCL2 binding to mGEnC-1 was evaluated with and without TNF-α activation The untreated condition is set at 100% (B). TNF-α-activated mGEnC-1 monolayers were treated with a cocktail of heparinases I, II and III, and binding of CXCL1, CXCL2 and CCL2 was analyzed by flow cytometry. The untreated condition is set at 100% (C). * P < 0.05 vs Untreated, *** P < 0.001 vs Untreated. (OD 450 nm, optical density at 450 nm).

We have previously used a mouse glomerular endothelial cell line (mGEnC-1) to determine that specific HS domains in the glomerular endothelial glycocalyx, which are increasingly expressed in response to inflammatory stimuli, and which facilitate leukocyte adhesion to the endothelial cell surface *in vitro*. Here, we evaluated the HS-dependent binding of chemokines CXCL1, CXCL2 and CCL2 to mGEnC-1. Incubation of TNF-α-activated mGEnC-1 with increasing concentrations of recombinant murine CXCL1, CXCL2 and CCL2 resulted in a dose-dependent increase in binding of the chemokines with 50%-effective concentrations (EC50) of 675–1100 ng/mL (CXCL1: 503–845 ng/ml, CXCL2: 654–1079 ng/ml, CCL2: 885–1328 ng/ml (95% confidence interval (CI)), Fig 1A). Stimulating mGEnC-1 with TNF-α appeared to increase binding of CXCL1, CXCL2 and CCL2 to the endothelial cell surface, although the effect was only significant for CCL2 (Fig 1B). Incubation with heparinases I, II and III significantly reduced the amount of HS in the mGEnC-1 glycocalyx, as evidenced by a decrease in binding of the anti-HS antibody AO4B08 (Fig 1C). HS removal reduced binding of CXCL1, CXCL2 and CCL2 to TNF-α-activated mGEnC-1 about 2-fold, indicating that HS in the glomerular endothelial glycocalyx contributes significantly to chemokine binding.

### CXCL1, CXCL2 and CCL2 binding to mGEnC-1 cells is differentially mediated by the sulfated GAGs HS and CS

**Fig 2:**
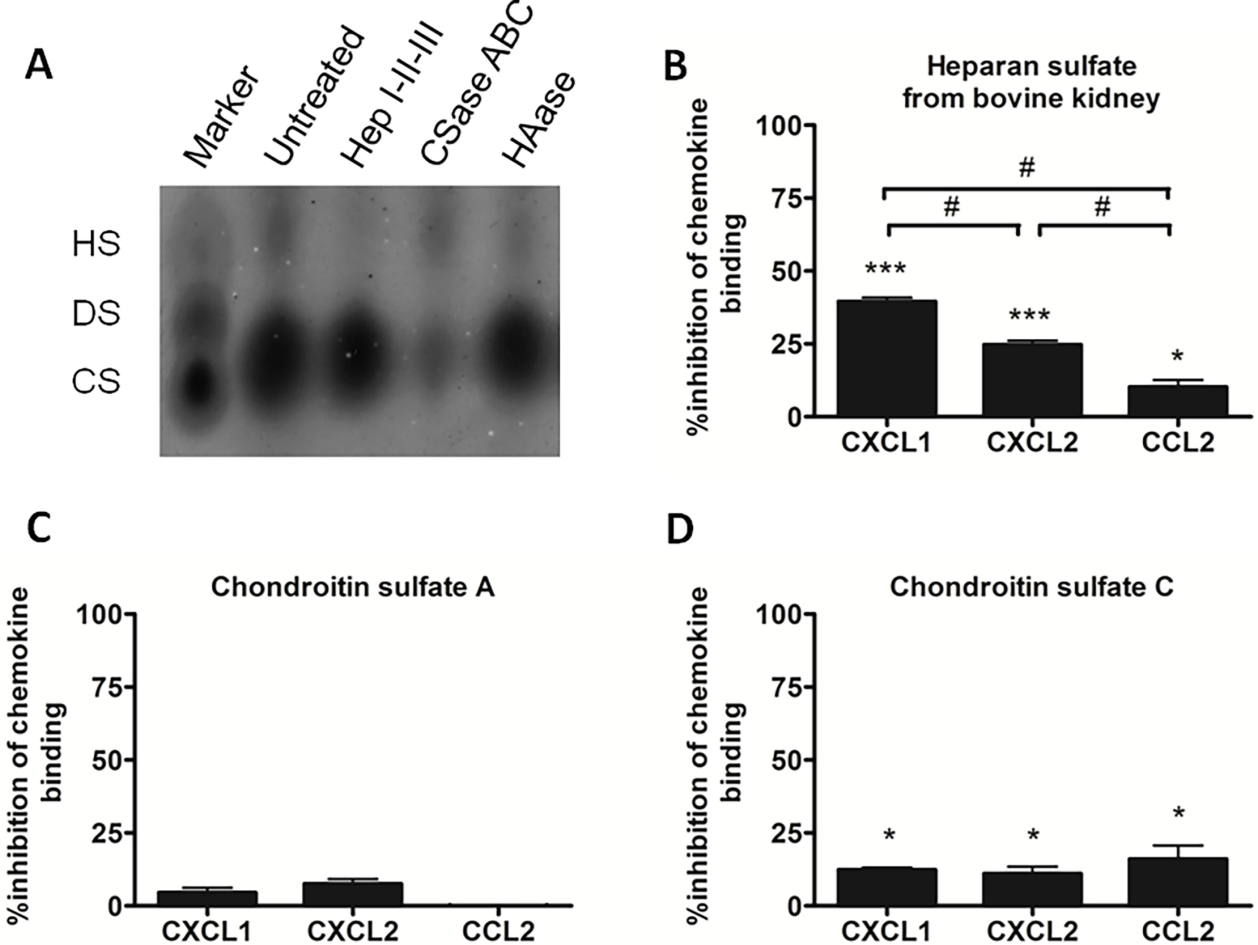
Sulfated GAGs HS and CS differentially contribute to CXCL1, CXCL2 and CCL2 binding to mGEnC-1. mGEnC-1 glycocalyx was isolated and visualized on barium acetate agarose gels. Treatment with GAG-specific glycosidases to confirm the identity of the observed GAG spots revealed that the mGEnC-1 glycocalyx consists primarily of HS and CS (A). Mouse recombinant chemokines CXCL1, CXCL2 and CCL2 were pre-incubated with heparan sulfate from bovine kidney (HSBK) (B), chondroitin sulfate A (CS-A) (C) and chondroitin sulfate C (CS-C) (D) (all used at 100 μg/ml) and added to TNF-α-activated mGEnC-1 in ELISA. Results are given as percentage inhibition of mGEnC-1 binding of the pre-incubated chemokines compared to chemokine binding in the absence of competing GAGs. * P < 0.05 vs control, *** P < 0.001 vs control, # P < 0.05.

Since degradation of HS decreases the negative charge of the mGEnC-1 glycocalyx, we determined whether binding of the basic chemokines is specifically mediated by HS compared to other sulfated GAGs in the mGEnC-1 glycocalyx. GAGs from mGEnC-1 cell cultures were visualized using barium acetate agarose gel electrophoresis and found to consist mainly of HS and CS, as confirmed by enzymatic digestion with heparinases or chondroitinases (Fig 2A). To evaluate if CS contributes to chemokine binding to mGEnC-1, CXCL1, CXCL2 or CCL2 were pre-incubated with HSBK, CS-A or CS-C (all used at 100 μg/ml) and binding to TNF-α-activated mGEnC-1 was evaluated in ELISA. Competition with HSBK resulted in 41% inhibition of CXCL1 binding to mGEnC-1, ~25% inhibition of CXCL2 binding and ~10% inhibition of CCL2 binding (Fig 2B). Pre-incubation with CS-A had no significant inhibitory effect on CXCL1, CXCL2 or CCL2 binding to mGEnC-1, whereas CS-C significantly reduced binding of CXCL1, CXCL2 and CCL2 by ~10–15% (Fig 2C and 2D).

### GAG preparations from different sources have distinct inhibitory effects on CXCL1, CXCL2 and CCL2 binding to mGEnC-1

**Fig 3:**
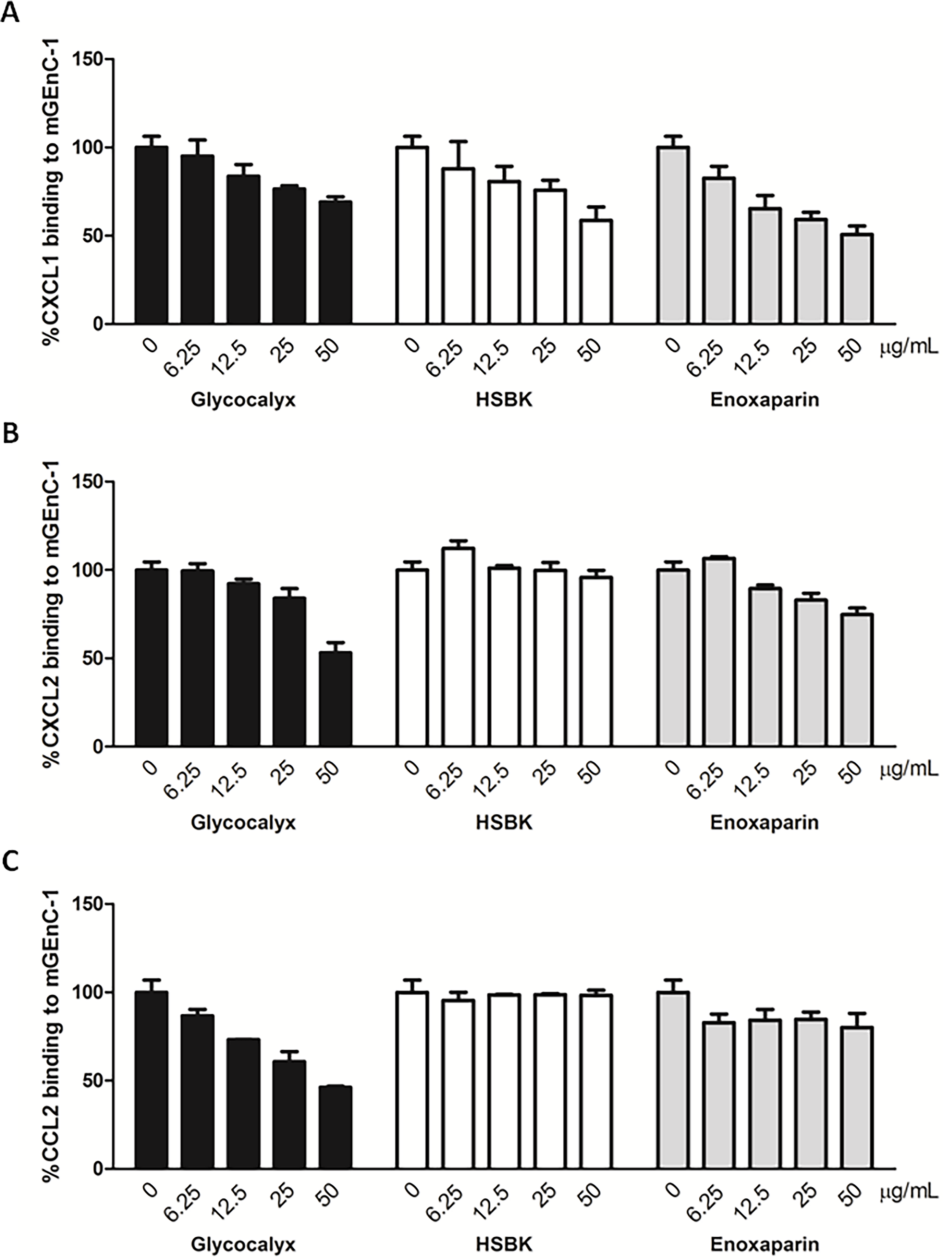
Chemokine binding to mGEnC-1 is differentially inhibited by mGEnC-1 glycocalyx, HSBK or enoxaparin. Mouse recombinant chemokines CXCL1 (A), CXCL2 (B) and CCL2 (C) were pre-incubated with serial dilutions (all ranging from 0 to 50 μg/ml) of mGEnC-1-derived glycocalyx, heparan sulfate from bovine kidney (HSBK) or highly sulfated low molecular weight heparin enoxaparin, and incubated with TNF-α-activated mGEnC-1 in ELISA. Results are given as percentage inhibition of mGEnC-1 binding of the chemokines pre-incubated with GAGs compared to chemokine binding in absence of competing GAGs.

Digesting HS in the mGEnC-1 glycocalyx comparably reduced binding of CXCL1, CXCL2 and CCL2, whereas HSBK inhibited chemokine binding to mGEnC-1 to varying degrees. Since HS is highly structurally heterogeneous, the inhibitory capacity of a GAG preparation will depend on the number of chemokine-binding HS domains it contains. Binding of CXCL1, CXCL2 and CCL2 to TNF-α-activated mGEnC-1 was therefore evaluated in competition with different GAG preparations, including isolated mGEnC-1-derived glycocalyx, HSBK or highly sulfated low molecular weight heparin (enoxaparin). Pre-incubating CXCL1 with serial dilutions of mGEnC-1-derievd glycocalyx, HSBK or enoxaparin dose-dependently reduced binding to mGEnC-1, with enoxaparin seeming to be the most effective competitor (Fig 3A). Binding of CXCL2 was most efficiently inhibited by isolated glycocalyx, followed by enoxaparin, whereas little inhibition was observed when pre-incubating with HSBK within the applied concentration range (Fig 3B). Finally, mGEnC-1-derived glycocalyx in turn inhibited CCL2 binding more than 2-fold, whereas CCL2 binding to mGEnC-1 was unaffected by competition with HSBK and only slightly reduced by pre-incubation with enoxaparin within the applied concentration ranges (Fig 3C). Together these results suggest that binding of CXCL1, CXCL2 and CCL2 to TNF-α-activated mGEnC-1 is mediated by specific HS domains that are differentially present within the different GAG preparations.

### Blocking specific HS domains in the mGEnC-1 glycocalyx with scFv anti-HS antibodies differentially inhibits chemokine binding

**Fig 4:**
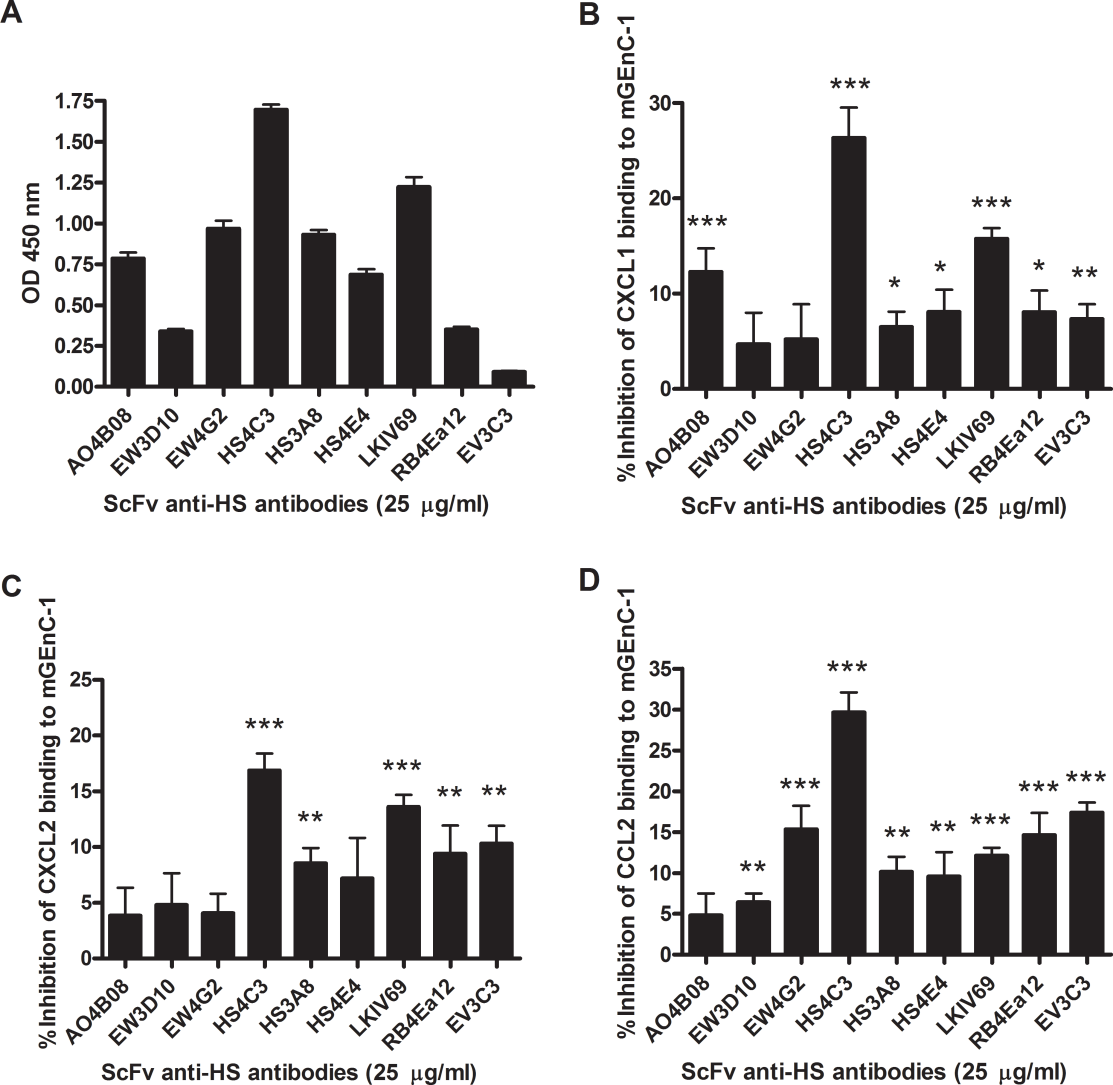
CXCL1, CXCL2 and CCL2 binding to HS on mGEnC-1 is differentially inhibited by anti-HS antibodies that recognize specific HS domains. mGEnC-1 were pre-incubated with single-chain variable fragment (scFv) anti-HS antibodies that recognize specific domains in HS chains before incubation with CXCL1, CXCL2 and CCL2. Differences in binding signals indicate the variable presence of specific HS domains recognized by the different antibodies in the mGEnC-1 glycocalyx (A). Competition results are depicted as percentage inhibition of chemokine binding to mGEnC-1 compared to binding to untreated cells. CXCL1 (B), CXCL2 (C) and CCL2 (D) binding to mGEnC-1 was differentially inhibited by pre-incubation with the specific scFv anti-HS antibodies. * P < 0.05 vs control, ** P < 0.01 vs control, *** P < 0.001 vs control.

We have previously identified scFv anti-HS antibodies that inhibit the interaction between neutrophils and TNF-α-activated mGEnC-1 by blocking specific HS domains in the mGEnC-1 glycocalyx. To determine the role of these and other HS domains in binding of the neutrophil-attracting chemokines CXCL1, CXCL2 and CCL2, TNF-α-activated mGEnC-1 were pre-incubated with scFv anti-HS antibodies before evaluating chemokine binding. ScFv anti-HS antibodies differentially bound TNF-α-activated mGEnC-1, suggesting variable expression of different HS domains in the mGEnC-1 glycocalyx (Fig 4A). CXCL1 binding to mGEnC-1 was significantly inhibited by all anti-HS antibodies except EW3D10 and EW4G2, with HS4C3, LKIV69 and AO4B08 being the most effective inhibitors (Fig 4B). Competition with anti-HS antibodies similarly reduced binding of CXCL2 to mGEnC-1, with the exception of AO4B08, which had no significant effect on CXCL2 binding (Fig 4C). CCL2 binding was significantly inhibited by all of the scFv anti-HS antibodies except AO4B08 (Fig 4D). Similarly to the results for CXCL1 and CXCL2, HS4C3 was the most effective inhibitor of CCL2 binding. In contrast, the antibodies EW4G2, RB4Ea12 and EV3C3 were more efficient in inhibiting CCL2 binding to mGEnC-1 compared to CXCL1 and CXCL2. Blocking HS in the mGEnC-1 glycocalyx using different anti-HS antibodies therefore suggests that binding of CXCL1, CXCL2 and CCL2 to mGEnC-1 is differentially mediated by specific HS domains.

### CXCL1, CXCL2 and CCL2 do not compete for the same binding sites on mGEnC-1

**Fig 5:**
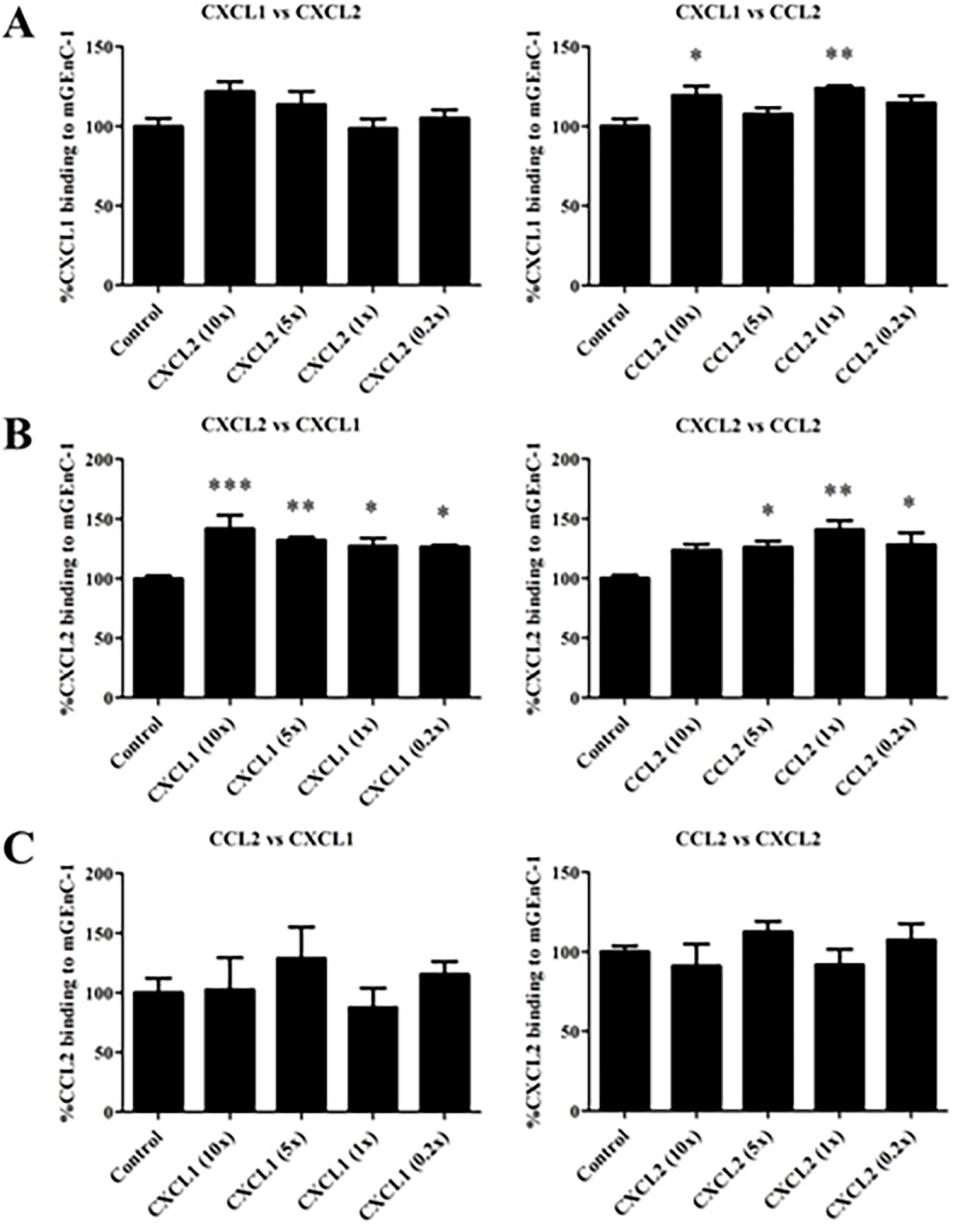
CXCL1, CXCL2 and CCL2 do not inhibit each other from binding to mGEnC-1. TNF-α-activated mGEnC-1 were pre-incubated with different chemokine concentrations (10, 5, 1 and 0.2-fold excess) before evaluating binding of the other chemokines in ELISA (CXCL1 detection (A), CXCL2 detection (B) and CCL2 detection (C)). Chemokine binding in absence of competing chemokine (Control) was set at 100%. * P < 0.05 vs control, ** P < 0.01 vs control, *** P < 0.001 vs control.

To confirm the chemokines’ specificity for distinct HS domains as suggested by the anti-HS antibody competition ELISAs, further competition assays were performed to determine if CXCL1, CXCL2 and CCL2 are able to block each other from binding to mGEnC-1 (Fig 5). For certain chemokine pairs, binding of the blocking chemokine appeared to facilitate the interaction between the other chemokines and mGEnC-1, e.g. CXCL2 versus CXCL1. However, none of the competing chemokines was able to reduce concentration-dependently the binding of the other two chemokines. Even when used at 10-fold excess (7.5 μg/ml), i.e. well within the range of the chemokines’ saturation concentrations, no inhibition was observed, suggesting that the endothelial glycocalyx can be saturated with and provide chemotactic stimuli from multiple chemokines simultaneously during inflammation.

## Discussion

The importance of specific HS domains for mediating leukocyte trafficking has been shown previously to depend on the degree of sulfation and distribution of sulfated domains along the HS chain. In particular, N- and 6-O-sulfated HS domains were increasingly expressed on inflammation activated glomerular endothelium and significantly enhanced leukocyte adhesion *in vitro* [6, 20].The same inflammation-promoting HS domains are expressed in glomerulonephritis *in vivo* [21, 34]. We therefore hypothesized that specific sulfated HS domains in the glomerular endothelial glycocalyx mediate binding of specific pro-inflammatory chemokines.

In the current study we determined that HS mediates binding of chemokines CXCL1, CXCL2 and CCL2 to mGEnC-1, supporting previous results that describe a HS/heparin dependence of CXCL1, CXCL2 and CCL2 binding and signaling [17–19, 35–37]. Digestion of cell surface HS comparably reduced binding of all chemokines, whereas competition with HSBK differentially inhibited the interaction between chemokines and the mGEnC-1 glycocalyx. While HSBK at high concentrations significantly inhibited binding of CCL2, the inhibitory effect was small compared to CXCL1 or CXCL2, suggesting that CCL2-binding HS domains are underrepresented in the HSBK preparation. CS-A, in which the hexosamine rings are 4-O-sulfated, a modification that is not found in HS, at high concentrations had no significant effect on chemokine binding. In turn, the 6-O-sulfation of CS-C might mimic 6-O-sulfation in HS, potentially explaining its observed, but weak, inhibitory capacity on chemokine binding.

Since the source of the HS preparation appeared to determine its inhibitory activity, competition assays were performed using isolated endothelial glycocalyx, HS from bovine kidney or the highly sulfated enoxaparin. Isolated glycocalyx inhibited binding of all chemokines, as it contains the same HS domains that mediate chemokine binding to mGEnC-1, illustrating the therapeutic potential of glycocalyx-derived glycosaminoglycan structures for reducing inflammation. Enoxaparin efficiently inhibited binding of CXCL1 and CXCL2, suggesting that these chemokines recognize domains with high levels of sulfation. HSBK in turn appears to be particularly rich in CXCL1-binding HS domains. As we previously showed that competition with enoxaparin or HSBK could inhibit neutrophil adhesion to activated mGEnC-1, the current results suggest that the decrease in leukocyte binding is accompanied by a decrease in chemokine binding to the mGEnC-1 glycocalyx.

The differential inhibition of CXCL1, CXCL2 and CCL2 binding to mGEnC-1 by the different GAG preparations illustrates that choosing the correct chemokine-binding HS domain(s) could enable selective inhibition of that chemokine. GAG mimetics, i.e. small molecules with structural characteristics similar to GAGs, including HS, are already explored as potential inhibitors of chemokine-HS interactions. For example, chlorite-oxidized oxyamylose (COAM) reduced the neutrophil recruitment/extravasation after peritoneal LPS injection in mice [38]. However, COAM was shown to bind CXCL1, CXCL2, CXCL6, CXCL10, CXCL11 and CCL5, but had no affinity for CCL2, CCL3 and CCL4 [38, 39], indicating that the observed inhibition is of low specificity. In contrast, we propose that the use of structurally defined HS domains may provide more potent and specific inhibitors for chemokine binding and activity.

A panel of scFv anti-HS antibodies was therefore used to investigate specific HS domains involved in CXCL1, CXCL2 and CCL2 binding. These antibodies have previously been utilized to determine the differential expression of HS domains in different tissues [28], as well as to identify HS domains important for leukocyte adhesion to glomerular endothelium *in vitro* and *in vivo* [6]. While antibodies EW3D10 and EW4G2 significantly reduced neutrophil adhesion to mGEnC-1, they did not inhibit binding of the neutrophil-attracting chemokines CXCL1 and CXCL2, suggesting that their HS domains might be ligands for cellular adhesion molecules rather than chemokines. In turn, HS4C3 previously had no effect on neutrophil adhesion, but efficiently blocked binding of all three tested chemokines to mGEnC-1, with a slight preference for CXCL1 and CCL2. Notably, HS4C3, and to a lesser extent LKIV69, also showed the strongest binding to mGEnC-1, suggesting that their corresponding HS domains are abundantly expressed in the mGEnC-1 glycocalyx, and might affect binding of chemokines to HS domains in close proximity as well. Accordingly, most of the anti-HS antibodies weakly inhibited chemokine binding to mGEnC-1 by 5–10%. However, several antibodies revealed interesting differences in their ability to compete with CXCL1, CXCL2 and CCL2 for HS domains in the mGEnC-1 glycocalyx. The 2-O-sulfated, IdoA-containing HS domain recognized by EV3C3 (Table 1) seems to at least overlap with the CCL2-binding HS domain, as it significantly inhibits chemokine binding by >15%, despite low overall binding to the mGEnC-1 glycocalyx. Competition with EW4G2 selectively inhibited binding of CCL2 by ~15%, but not CXCL1 or CXCL2 as well. In turn, competition with antibody AO4B08 significantly reduced binding of CXCL1, but not CXCL2 or CCL2. The absence of competition between the different chemokines for binding sites on mGEnC-1 further indicates specificity in their interaction with cell surface ligands. Furthermore, the lack of competition suggests that the endothelial glycocalyx can simultaneously integrate chemotactic stimuli for different leukocytes during inflammation, even when saturated with chemokines. These findings point towards a degree of specificity in the interaction between GAGs and CXCL1, CXCL2 or CCL2, and raise the possibility of specific HS-based or anti-HS-based therapeutics to inhibit inflammatory processes. Limiting chemokine binding by blocking their specific binding site on HS may have strong functional implications, since most chemokines di- or oligomerize in the presence of HS for efficient chemotaxis, or presentation to chemokine receptors [19, 40–42].

Although our results indicate that specific HS domains regulate binding of the chemokines CXCL1, CXCL2 and CCL2 to the glomerular endothelial glycocalyx, information on the exact chemokine binding-HS domains is limited to the modifications and domains recognized by the array of scFv anti-HS antibodies. In-depth analysis of the glycocalyx, e.g. using high resolution chromatography combined with mass spectrometry, is required to structurally define HS domains which mediate binding of a specific chemokine. By combining our recently described method for isolating GAGs from biological samples with such GAG analysis techniques, these HS domains may be identified and lead to the development of GAG-based drugs that target specific chemokine-HS interactions, thereby enabling fine-tuning of inflammatory processes in the kidney and possibly other organs.
